# Methaneseleninic acid and γ-Tocopherol combination inhibits prostate tumor growth *in Vivo* in a xenograft mouse model

**DOI:** 10.18632/oncotarget.1979

**Published:** 2014-05-18

**Authors:** Chandra K. Singh, Mary A. Ndiaye, Imtiaz A. Siddiqui, Minakshi Nihal, Thomas Havighurst, KyungMann Kim, Weixiong Zhong, Hasan Mukhtar, Nihal Ahmad

**Affiliations:** ^1^ Department of Dermatology, University of Wisconsin, Madison, WI; ^2^ Department of Biostatistics and Medical Informatics, University of Wisconsin, Madison, WI; ^3^ Department of Pathology and Laboratory Medicine, University of Wisconsin, Madison, WI; ^4^ University of Wisconsin Carbone Cancer Center, Madison, WI

**Keywords:** SELECT, Selenium, Vitamin E, Prostate Cancer, Chemoprevention

## Abstract

Studies have shown that vitamin E and selenium possess antiproliferative effects against prostate cancer (PCa). However, results from the Selenium and Vitamin E Cancer Prevention Trial (SELECT) suggest that vitamin E (α-tocopheryl acetate; 400 mg) and/or selenium (L-selenomethionine; 200 μg) were ineffective against PCa in humans. It is arguable that the selected dose/formulation of vitamin E/selenium were not optimal in SELECT. Thus, additional studies are needed to define the appropriate formulations/dose regimens of these agents. Here, we investigated the effect of methaneseleninic acid (MSA; 41 μg/kg) and/or γ-tocopherol (γT; 20.8 mg/kg or 41.7 mg/kg) in Nu/J mice implanted with 22Rν1 tumors. MSA (41 μg/kg) and γT (20.8 mg/kg) combination was most consistent in imparting anti-proliferative response; resulting in a significant decrease in i) tumor volume/weight, ii) serum PSA, and iii) Ki-67 immunostaining. Further, we observed i) an upregulation of pro-apoptosis Bax and a down-regulation of the pro-survival Bcl2, and ii) an increase in pro-apoptosis Bad. Furthermore, the combination resulted in a modulation of apolipoprotein E, selenoprotein P and Nrf2 in a fashion that favors antiproliferative responses. Overall, our study suggested that a combination of MSA and γT, at lower dose regimen, could be useful in PCa management.

## INTRODUCTION

PCa is a major health problem in elderly males. In the United States alone, 238,590 new PCa cases and 29,720 related deaths were expected in the year 2013 [1]. The impact of PCa on public health has spawned tremendous interest in the possible use of non-toxic chemopreventive agents for prevention as well as treatment of this neoplasm. A wide range of naturally occurring chemopreventive agents have been tested for PCa management in chemopreventive as well as therapeutic settings. Selenium and vitamin E are essential human dietary components, which have been shown to possess therapeutic and preventive effects against PCa in preclinical, epidemiological, and phase III randomized placebo-controlled clinical trials [2-4]. Based on these studies, the Selenium and Vitamin E Cancer Prevention Trial (SELECT) was designed and initiated in the year 2001 to test the hypothesis that daily use of selenium (in the form of 200 μg of L-selenomethionine) and vitamin E (in the form of 400 mg of all rac α-tocopheryl acetate), used alone or in combination, would prevent PCa in a cohort of 35,533 healthy men who were at average risk for PCa [5]. In 2008, SELECT was prematurely terminated after 5.5 years (1.5 years before its intended minimum follow up length) because selenium and vitamin E were not able to prevent PCa incidences [5]. Further, a significant increase in incidences of PCa was noticed in a longer follow-up study of SELECT participants [6]. Thus, the outcome of SELECT was disappointing for the scientific community as well as general public.

Interestingly, concerns were raised by the scientific community regarding the premature nature of the trial and the chosen formulations and doses of vitamin E and selenium used, even before the start of SELECT ([7], and reviewed in [8]). Indeed, according to the USDA Dietary Reference Intake Data, the Recommended Daily Allowances (RDA) for selenium is 55 μg (3.6 times lower dose than used in SELECT) and 15 mg for vitamin E (26.7 times lower dose than used in SELECT) [9]. In addition, SELECT was a prevention trial based on a long-term intake of the two agents. Therefore, arguments can be made against the used high dose regimens of selenium and vitamin E. This is particularly relevant to the dose of vitamin E because the design of SELECT was based on two previous clinical trials, 1) the Alpha-Tocopherol Beta Carotene Cancer Prevention (ATBC) study, and 2) the Nutritional Prevention of Cancer (NPC) study [4, 10]. While SELECT used the same formulation of vitamin E as in ATBC, the dose was 8 times higher (50 mg/day in ATBC vs. 400 mg/day in SELECT) [4]. Interestingly, another previous trial suggested that high-dose (≥400 mg/day) vitamin E supplements may in fact increase all-cause mortality and should be avoided [11]. Similarly, while the selenium dose remained the same in SELECT as in the NPC study (200 μg/day), SELECT used selenomethionine while NPC had used selenium-enriched yeast [12]. In both previous trials, the ages of the subjects were about 10 years higher than that those recruited in SELECT. In addition, the biology of vitamin E and selenium was not well-understood at the time when SELECT was designed.

Following the failure of SELECT, a number of formulations and dose regimens of vitamin E and selenium are being revisited in many pre-clinical studies. Some studies have suggested that while γ-tocopherol (γT) is effective against cancer, α-tocopherol (the formulation that was used in SELECT) lacks anti-proliferative effects in different cancer models [13-17]. Similar observations were made for selenomethionine, which was used in SELECT as the source of selenium [18-20]. One head-to-head study demonstrated superior growth inhibitory efficacy of methaneseleninic acid (MSA) over selenomethionine and selenite in human PCa xenograft models [18]. Another interesting study compared the effects of different selenium compounds on mouse prostate proteome profiles and suggested MSA to be the best among the four tested selenium compounds at preferentially altering the proteome [19]. Additionally, dietary supplementation with MSA, but not selenomethionine, was found to reduce spontaneous metastasis of Lewis lung carcinoma in C57BL/6 mice [20]. Thus, these recent studies provided us a rationale to determine the efficacy of MSA and/or γ-tocopherol against PCa in nude mice xenografts.

## RESULTS

### Effects of MSA and/or γT on 22Rν1 implanted prostate tumors

**Figure 1:**
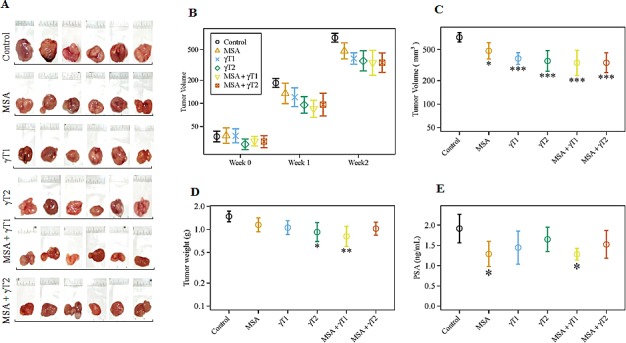
Effects of oral MSA and/or γT on prostate tumor growth in athymic nude mice Mice were subcutaneously implanted with 22Rν1 cells, and after 12 days MSA and/or γT were administered as described in ‘Materials and Methods’. Effects of MSA and/or γT were assessed for tumor size, tumor volume, tumor wet weight and serum PSA levels. Following sacrifice, tumors were resected. Representative images of each treatment group are shown (A). Tumor volume was measured at the start of the experiment and subsequently at 7^th^ and 14^th^ days after starting treatment (B). At the end of the experiment, tumor volume was determined (C); Average wet weights of resected tumors were taken (D); and serum PSA levels were measured (E). All are represented as mean value ± 2 standard errors of mean (*P<0.05, **P<0.01 and ***P<0.001).

This *in vivo* study was performed to assess the efficacy of MSA and γT alone and in combination in athymic nude mice implanted subcutaneously with human prostate carcinoma 22Rν1 cells. This cell line was established from a human prostate tumor xenograft (CWR22R) and shows characteristics of well differentiated adenocarcinoma [21]. An advantage of this cell line is that when implanted in nude mice, they release PSA that can be measured in blood. Our specific objectives were to determine, 1) if MSA or γT (at two different doses, shown as γT1 and γT2) alone possesses anti-proliferative effects against prostate tumorigenesis *in vivo*, and 2) whether two different combinations of these agents - MSA + γT1 and/or MSA + γT2 are better than either MSA or γT alone. The tumor data (tumor size, tumor volume and wet weight) and serum PSA levels are presented in figure 1. Representative tumor images of each treatment group are presented in figure 1A which clearly shows shrinking size of the tumor in all treatment groups but most predominantly in MSA + γT1 group. Although we found a decreasing trend in tumor volume after one week in all treatment groups (Figure 1B), a significant decrease in tumor volume was observed at the termination of study (Figure 1C). However, a significant decrease in tumor weight was only evident for γT2 and MSA + γT1 groups whereas the other three treatment groups showed a non-significant decreasing trend (Figure 1D). We also determined the effect of treatments on the prostate specific marker, PSA which is an indicator of PCa progression. Interestingly, although all the treatment groups showed a decreasing trend in serum PSA levels, when analyzed for statistical significance, only the MSA and MSA + γT1 groups demonstrated significance (Figure 1E).

### Effects of MSA and/or γT on proliferation index in 22Rν1 implanted prostate tumors

**Figure 2:**
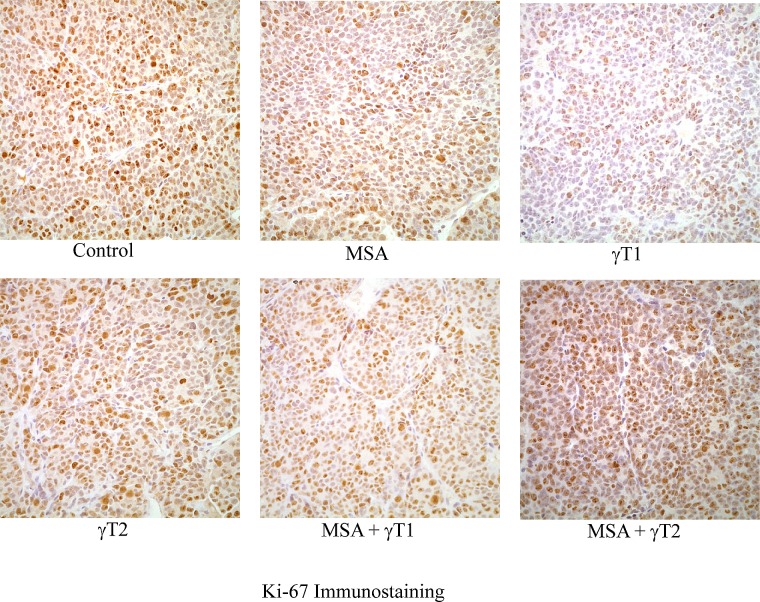
Effects of MSA and/or γT on tumor cell proliferation At the termination of xenograft experiment, tumors were excised and processed for Ki-67 immunostaining as detailed in ‘Materials and Methods’. Immunohistochemical analysis was performed in tumor tissue of control and treatment groups under bright field microscope. Representative images (400x magnification) of each treatment group are shown.

In the next experiment, we determined the effect of MSA and/or γT on proliferation in tumor tissues harvested from animals of different treatment groups. This was achieved by assessing the staining intensity of the proliferation marker Ki-67. As shown in figure 2, a decreasing trend in Ki-67 immunostaining was observed in all of the treatment groups. However, no experimental groups achieved statistical significance for Ki-67 intensity score (data not shown).

### Effects of MSA and/or γT on Bax/Bcl2 ratio (apoptosis index) in 22Rν1 implanted prostate tumors

**Figure 3:**
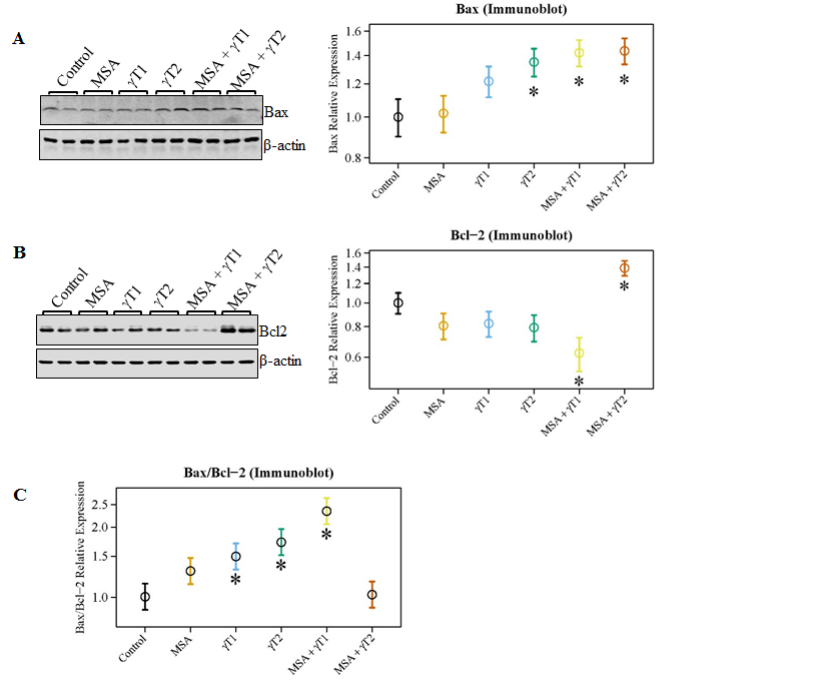
Effects of MSA and γT on Bax and Bcl2 proteins At the termination of the experiment, the effects of the treatments on Bax and Bcl2 proteins were determined using immunoblot analysis. 40 μg protein was separated on SDS-PAGE and immunobloted for Bax (A) and Bcl2 (B) as detailed in ‘Materials and Methods’. The blots were reprobed for β-actin for loading control. Representative blots are shown and the data (relative density normalized to β-actin) are expressed as mean ± 2 standard error of three replicates (representing 6 mice). Ratio of Bax/Bcl2 was also calculated and plotted (C) (*P<0.05).

Since a dysregulation of apoptosis machinery is a major characteristic of cancer growth, we next examined the effect of MSA and/or γT on protein levels of the apoptosis markers Bax and Bcl2 and calculated the apoptosis index by determining the ratio of pro-apoptosis Bax to anti-apoptosis Bcl2 (Bax/Bcl2). As shown in figure 3, we observed a significant increase in the pro-apoptosis protein Bax in the γT2, MSA + γT1 and MSA + γT2 groups; however, a significant decrease in the anti-apoptosis protein Bcl2 was only noticed in the MSA +γT1 group (Figure 3A and 3B). Surprisingly, the expression level of Bcl2 in the MSA + γT2 group was found to be even higher than that of control. This resulted in an apoptosis index (i.e. Bax/Bcl2 ratio) that was only significantly higher in the γT1, γT2 and MSA + γT1 groups at the protein level (Figure 3C).

**Figure 4:**
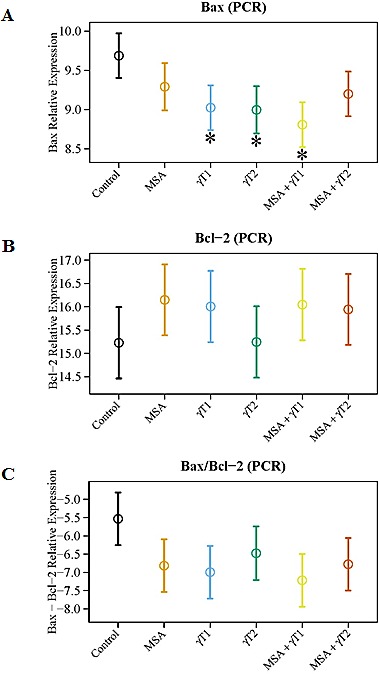
Effects of MSA and/or γT on Bax and Bcl2 mRNA At the termination of xenograft experiment, effects of treatments on Bax and Bcl2 transcription were assessed by qRT-PCR analyses. RNA from tumor tissue samples was isolated and cDNA was made. qRT-PCR was run for Bax and Bcl2 as detailed in ‘Materials and Methods’. GAPDH was used as endogenous control. The qRT-PCR data are represented as relative mRNA levels for Bax (A) and Bcl2 (B). Ratio of Bax/Bcl2 mRNA was calculated and plotted (C). The data represented are mean ± 2 standard error of three replicates (representing 6 mice) (*P<0.05).

We also determined the effects of treatments on Bax and Bcl2 modulation at the transcriptional level (Figure 4). As evident from the qRT-PCR data, compared to control, Bax was significantly up-regulated in γT1, γT2 and MSA + γT1 treatment groups, whereas Bcl2 expression was noticeably down-regulated in the MSA, γT1 and MSA + γT1 groups (Figure 4A and 4B). The qRT-PCR data demonstrated a marked increase in the Bax/Bcl2 ratio in MSA, γT1 and MSA + γT1 groups, as evident form decreasing ΔCT value (Figure 4C). The qRT-PCR data are plotted as ΔCT, means decrease value indicate increase in gene expression and *vice-versa*.

### Effects of MSA and/or γT on pro-apoptosis Bad in 22Rν1 implanted prostate tumors

**Figure 5:**
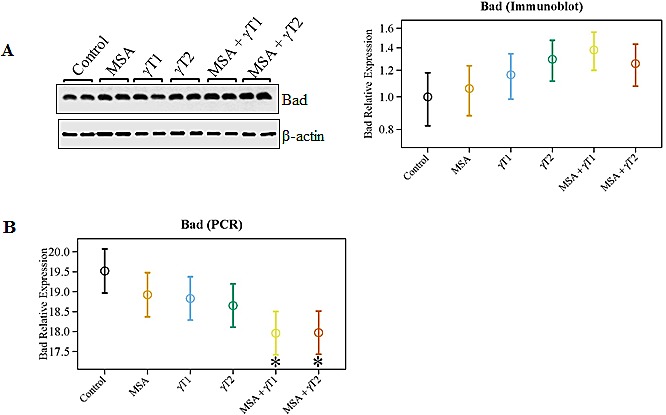
Effects of MSA and/or γT on Bad protein and mRNA The effects of MSA and γT on pro-apoptosis Bad was analyzed at protein as well as mRNA levels. For immunoblot analysis, 40 μg protein was separated on SDS-PAGE and immunobloted as detailed in ‘Materials and Methods’. The blots were reprobed for β-actin for loading control. Relative protein expression of Bad was calculated and plotted (A). Relative mRNA levels of Bad were assessed using qRT-PCR assay as detailed in ‘Materials and Methods’ (B). The data represented are mean ± 2 standard error of three replicates (representing 6 mice) (*P<0.05).

Another Bcl2 family protein, Bad, is known to play an important role in apoptosis induction. Therefore, we analyzed the effects on pro-apoptosis Bad at protein as well as mRNA levels. As shown in figure 5A, marked increase were observed in Bad protein levels in the γT2 and MSA + γT1 group. In addition, at the mRNA levels, we observed a trend of increased Bad level in all the treatment groups; however, a significant increase was found only in MSA + γT1 and MSA + γT2 groups (Figure 5B).

### Effects of MSA and/or γT on ApoE, SepP and Nrf2 in 22Rν1 implanted prostate tumors

**Figure 6:**
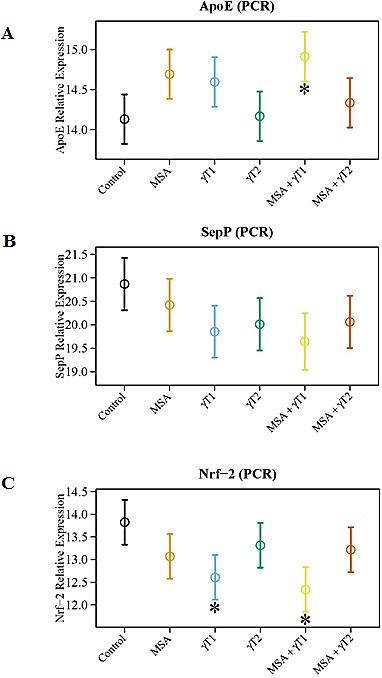
Effects of MSA and/or γT on oxidative stress markers To assess the effects of MSA and/or γT on modulation of oxidative stress, ApoE, SepP and Nrf2 mRNA level were analyzed. RNA isolation from tumor tissue followed by cDNA synthesis and then qRT-PCR was performed as detailed in ‘Materials and Methods’. The qRT-PCR data are represented as relative quantity (normalized to GAPDH) for ApoE (A), SepP (B) and Nrf2 (C) transcriptional levels. Representative data are expressed as mean ± 2 standard error of three replicates (representing 6 mice) (*P<0.05).

Since cellular oxidative stress plays a critical role in determining the ultimate fate of a cell, we determined the effects of MSA and/or γT on several important oxidative stress regulators (*viz*. ApoE, SepP and Nrf2), which have relevance to human PCa. An increased expression of apolipoprotein E (ApoE) has been correlated directly with Gleason score in human PCa tissues, hormone independence as well as local and distant invasiveness [22]. Further, in a separate study, increased concentrations of plasma cholesterol and ApoE were shown in rats with diets deficient in selenium and vitamin E [23]. With this in mind, we determined the effects of MSA and/or γT on the expression level of ApoE. We found a significant reduction in mRNA levels of ApoE in MSA + γT1 group only. However, a decreasing trend was evident in MSA and γT1 groups. No change were noticed in the ApoE levels in γT2 and MSA + γT2 groups (Figure 6A).

Selenoprotein P (SepP) is the central protein in blood that controls selenium transport and distribution, and is responsible for the extracellular antioxidant defense properties of selenium. A recent study showed a downregulation of SepP in PCa which leads to decreased cellular protection against oxidative damage [24]. Our data demonstrated that compared to control, a marked up-regulation of SepP occurs in all the treatment groups except MSA (Figure 6B). However, the low dose of γ-tocopherol (γT1 group) and its combination with MSA (the MSA + γT1 group) was seemingly found to have the best response among all the treatment groups.

One of the key pathways that cells use in the antioxidant response to oxidative stress is headed by nuclear factor-erythroid 2-related factor 2 (Nrf2). As MSA and γ-tocopherol are both antioxidants, it makes sense that these would affect levels of Nrf2. Down-regulation of the transcription factor Nrf2 has been reported in the transgenic adenocarcinoma of the mouse prostate (TRAMP) model of PCa [25]. This information led us to investigate the effects of MSA and γT on Nrf2 expression level. As shown in figure 6C, we found a significant up-regulation in Nrf2 levels in γT1 and MSA + γT1 treatment groups only.

Taking all of the experimental data into consideration, a clearly evident finding of our study is that among the different treatment groups tested, the MSA + γT1 group (MSA in combination with low dose of γ-tocopherol) showed the most consistent and favorable tumor inhibitory response.

## DISCUSSION

The negative findings of SELECT were disappointing and puzzling to many scientists, even though selenium and vitamin E showed great promise in treating PCa. Both of these agents have been shown to possess anti-proliferative effects in a number of models (reviewed in [8, 26]). In a recent review, we discussed the studies done prior to and since the inception of SELECT, as well as the parameters of the trial itself [8]. In this review, based on a wealth of available data, we provided arguments to study the most effective doses and formulations of selenium and vitamin E for their efficacy against PCa [8]. The objective of our current investigation was to ascertain the efficacy of two specific formulations of selenium and vitamin E, MSA and γT, respectively, against PCa *in vivo.* The rationale for selecting these formulations was based on recent studies showing better efficacy of γT over α-tocopherol [13-17] and MSA over selenomethionine or selenite [18-20].

Similarly, the selected doses were also based on published animal studies and human clinical trials. The dose regimen used in our study corresponded to a human equivalent daily dose of 142 μg/day for MSA and 72 or 142 mg/day for γT (based on 7 days/week), which is significantly lower than those used in SELECT. The conversion of doses between the two species is based on calculations described elsewhere [27]. It is important to mention here that unlike SELECT, which was a prevention trial, our preclinical study represents an intervention trial.

Under the experimental conditions employed, we found that compared to control, all the treatment groups showed significant decreases in tumor volumes. However, the tumor weight was significantly affected only in the γT2 and MSA + γT1 groups. We also found that only the MSA and MSA + γT1 groups demonstrated significant decreases in the serum levels of PSA. Thus, the MSA + γT1 group that consists of MSA and the low dose of γ-tocopherol demonstrated the most consistent response among all the treatment groups. Interestingly, the combination with the high γ-tocopherol dose (MSA + γT2 group) was not any better that the combination with low dose γ-tocopherol (MSA + γT1 group), and in some cases had less favorable outcomes. Since there is an established correlation between Ki-67 levels and the histopathological grade of neoplasms [28], we also assessed the effects of MSA and/or γT treatments on Ki-67 staining in tumor tissues. We found a decreasing trend of Ki-67 immunostaining with all treatments. However, because of large variation among individual samples, it was not possible to reach any meaningful conclusion in this qualitative analysis. Thus, the tumor data together with PSA and Ki-67 data suggest that a higher dose of γ-tocopherol may not be desirable. This is also supported by a published study showing null results for PCa incidence in the rat prostate treated with relatively higher doses of selenium and/or vitamin E in the form of l-selenomethionine and dl-α-tocopherol [29].

We were also interested in determining the correlative involvement of Bcl2 family proteins in the anti-proliferative response of MSA and/or γT in prostate tumors. The rationale for determining the effects on Bcl2 family proteins is based on several studies showing that they are differentially expressed in various malignancies and are considered as useful prognostic biomarkers [30-32]. In addition, we have earlier demonstrated that a combination of MSA and +-alpha-tocopheryl succinate enhances Bax/Bcl2 ratio and causes apoptosis of human PCa cells in culture [30]. It is known that Bcl2 acts as a pro-survival protein, whereas Bax and Bad have pro-apoptosis function. We found a significant increase in Bax levels in γT2, MSA + γT1 and MSA + γT2 groups, whereas Bcl2 levels were significantly down-regulated in the MSA + γT1 group. Interestingly, Bcl2 was found to be significantly up-regulated in MSA + γT2 (MSA in combination with high dose of γ-tocopherol) group. Further, we also found that only the MSA + γT1 tumors shared a significant increase in the Bax/Bcl2 ratio and enhanced pro-apoptosis Bad levels. These are important observations because the Bax/Bcl2 ratio as well as Bad dimerization with Bcl2 has been shown to play a significant role in deciding the fate of a cell (death v*ersus* survival) [30, 33].

As a final part of our study, we evaluated the effects of MSA and/or γT on oxidative stress related markers because oxidative stress plays a major role in PCa initiation and progression [34, 35] and both MSA and γT are known antioxidants. We determined the effect of MSA and/or γT on ApoE, which is the main apoprotein of chylomicrons and is essential for the normal catabolism of triglyceride-rich lipoprotein constituents. ApoE mediates the binding, internalization, and catabolism of lipoprotein particles and increased levels of ApoE have been noticed in PCa tissues as well as in rats maintained on selenium and vitamin E deficient diets [22, 23]. It has been shown that the prostatic intraepithelial neoplasias (PINs) adjacent to clinically manifested cancer are positive for ApoE, whereas more distant PINs are not, suggesting the relevance of ApoE as a marker of aggressiveness in human PCa [22]. Thus, our data showing a significant transcriptional down-regulation of ApoE in the MSA + γT1 groups is relevant to PCa.

We also analyzed the effects of treatments on modulations in SepP, which is used by cells for selenium transport, thereby allowing the cell to make use of the antioxidative capacity of selenium. A recent study demonstrated that SepP is reduced in 60.8% of human prostate tumors as compared to benign prostates [24]. Our data shows marked increase in SepP expression in γT1 and MSA + γT1 groups, favoring lower dose of γT over a higher dose. Finally, we determined the effects of MSA and γT on Nrf2, an important transcription factor that plays a major role in maintaining cellular redox homeostasis [36]. Nrf2 activators are being appreciated as possible pharmacological agents for PCa management. Selenium compounds have been shown to increase Nrf2 in PCa cells [37]. Further, Huang et al. have shown that Nrf2 is epigenetically suppressed due to CpG hypermethylation in TRAMP mouse prostate tumors [25]. Studies have shown that dietary feeding of a γ-tocopherol-rich mixture of tocopherols suppressed prostate tumorigenesis in TRAMP mice, and that this was associated with higher Nrf2 levels [17, 25]. In our study, we have found a significant increase in Nrf2 in γT1 and MSA + γT1 treatment groups, suggesting a favorable redox-modulation by these agents.

Taken together our data suggest that MSA and γT are possibly better formulations of selenium and vitamin E and lower dose of γT may be more useful than the high dose regimens. This is especially important in view of the fact that the extended follow-up of SELECT suggested an association between vitamin E (high dose) intake and PCa incidence [6]. We firmly believe that low doses of γT as well as MSA could be beneficial for PCa and possibly other redox associated conditions. The low dose regimens (at or slightly above the *recommended daily allowance* levels) of vitamin E as well as selenium (in the form γT and MSA) may be most useful in cancer control in prevention settings, where a continuous supplementation could be useful in maintaining a redox balance in the body. We suggest that further detailed preclinical studies are needed to select the best possible formulations and doses of selenium and vitamin E for PCa management, both in prevention as well as intervention settings. In this direction an extensive preclinical study in a human relevant mouse model of PCa, such as Hi-Myc and/or Pten deficient models, could be a step forward towards future human clinical trials with these agents.

## MATERIALS AND METHODS

### Preparation of compounds

Methaneseleninic acid (MSA) and γ-tocopherol (γT) were purchased from Sigma-Aldrich Co. (Catalog numbers 541281 and T1782, respectively). Stock solution of MSA was prepared at a concentration of 645 μM in deionized water, filter sterilized, aliquoted into 1 mL portions, and frozen at −20°C. A new stock was thawed daily for use. Stock solutions of γT were prepared at two concentrations, 100 mM and 200 mM, in tocopherol-stripped corn oil (Dyets, Inc.), aliquoted, and stored in the dark at 4°C.

### Cell culture

The human PCa cell line 22Rν1 was obtained from the American Type Culture Collection (ATCC) and cultured in RPMI-1640 (Mediatech, Inc.) supplemented with 10% FBS (Sigma-Aldrich Co.) under standard cell culture conditions (37°C, 5% CO_2_ in a humidified incubator). The cells used in our experiments were purchased within 6 months from the start of the experiments and were characterized at the ATCC via observation and identity confirmed by Short Tandem Repeat (STR) analysis.

### Nude mouse xenograft experiments

Housing and care of the animals was approved by the University of Wisconsin Institutional Animal Care and Use Committee in accordance with the NIH Guidelines for the Care and Use of Laboratory Animals. Eight week old male Nu/J mice (The Jackson Laboratories, stock number 2019) were maintained at the Association for Assessment and Accreditation of Laboratory Animal Care (AAALAC)-accredited Laboratory Animal Resource Facility at the University of Wisconsin at standard conditions (in microisolator cages under pathogen-free conditions with 12 h light/12 h dark schedule). The animals had *ad libitum* access to irradiated diet (Catalog number 7912, Harlan Teklad, Madison, WI) and water.

For implanting 22Rν1 tumors, the confluent cells were harvested via trypsinization and resuspended in RPMI-1640 serum free medium, and kept on ice until injections. Just prior to implantation, 1x10^6^ cells were mixed with Matrigel (BD Biosciences) at a 1:1 ratio and subcutaneously injected into the right flank of each mouse. The tumors were allowed to grow for 12 days, and the mice were then divided into 6 groups (consisting 10 animals in each) keeping average baseline tumor volume (~36 mm^3^) same for each experimental group. The mice were administered methaneseleninic acid and/or γ-tocopherol via oral gavage 5 days/week for 2 weeks as described by Li et al. [18] employing the following protocol: Group 1: control (vehicle alone; tocopherol-stripped corn oil plus sterile water, 15 μL each); Group 2: MSA (40.95 μg/kg body weight in 15 μL sterile water); Group 3: γT1 (γ-tocopherol, dose 1; 20.83 mg/kg body weight in 15 μL corn oil); Group 4: γT2 (γ-tocopherol, dose 2; 41.66 mg/kg body weight in 15 μL corn oil); Group 5: MSA + γT1; Group 6: MSA + γT2. Tumor volume was measured weekly with a Vernier caliper using the formula (π/6 × Length × Width × Height). The study was terminated on 14^th^ day from the treatment start date. At this point, the mice were sacrificed, blood was collected for prostate specific antigen (PSA) analysis, and tumors were resected, photographed, weighed, and either flash-frozen for further protein and mRNA analysis, or fixed in formalin for paraffin embedding and sectioning.

### Immunohistochemical analysis of Ki-67

The formalin fixed tissue specimens were processed, embedded into paraffin, and sectioned at the University of Wisconsin Carbone Cancer Center (UWCCC) Experimental Pathology Core Laboratory. The slides were deparaffinized using xylenes and graded ethanol followed by antigen retrieval using IHC-Tek epitope retrieval steamer set (IHC World, LLC.). Slides were then blocked with 10% goat serum, incubated with primary antibody against Ki-67 (Santa Cruz Biotechnology, Inc.) followed by anti-rabbit secondary antibody, and then detected using DAB (3, 3'- diaminobenzidine) peroxidase substrate kit (Vector Labs, Inc.). The tissue sections were counterstained with Harris modified hematoxylin (Thermo Fisher Scientific Inc.), dehydrated with graded ethanol and xylene, and mounted with paramount diluted with xylene (1:2 ratio). For images and intensity scoring, a trinocular bright field Olympus BX41 microscope (Olympus Corp. of the Americas) was used. Tissue cores were scored as negative (0), weak (+), moderate (++), or strong (+++) by staining intensity.

### Immunoblot analysis

A small amount of tumor tissue (~100 mg) was homogenized in 1X RIPA buffer (EMD Millipore Corp.) containing 10 μL/mL protease inhibitor cocktail (Thermo Fisher Scientific Inc.) and 1 mM PMSF (Amresco, LLC) using an Ultra-Turrax T25 Basic homogenizer (IKA Works, Inc.). Protein was quantified using the BCA Protein Assay Kit (Thermo Fisher Scientific Inc.). For immunoblot analysis, equal amounts of proteins from three individual tumors were pooled together to give one sample and 40 μg of pooled protein was subjected to SDS-PAGE, transferred onto a nitrocellulose membrane and blocked with 5% non-fat dry milk. The membrane was then probed with primary antibodies against Bax, Bcl2 or Bad (Santa Cruz Biotechnology Inc.) followed by an appropriate secondary HRP-conjugated antibody. The antigen-antibody complex was developed using chemiluminescence detection (Thermo Fisher Scientific Inc.), and images were captured and densitometry analysis was performed using the Kodak Image Station 4000MM (Carestream Health, Inc.).

### Assay for PSA measurement

Quantitative measurement of PSA was performed in serum samples of mice using the PSA (Human) enzyme-linked immunosorbent assay (ELISA) Kit (Abnova Corp.) according to manufacturer's protocol. The assay system uses a goat anti-PSA antibody directed against PSA for solid phase immobilization. The serum samples were allowed to react and the color intensity of the developed reaction was measured at 450 nm using a Multiskan MCC/340 micro-plate reader (Thermo Fisher Scientific Inc.).

### Quantitative real time reverse transcription PCR (qRT-PCR) analysis

RNA was extracted from tumor tissue using QIAshredder and RNeasy Mini Kit (Qiagen) and first strand cDNA was transcribed with random primers, dNTPs and M-MLV reverse transcriptase (Promega). For qRT-PCR analysis, equal amount of cDNA from two individual tumors were pooled together to give one sample, and total of three samples (representing 6 mice) were analyzed. qRT-PCR was performed with SYBR Premix Ex Taq II (Clontech Laboratories, Inc.) with pooled first strand cDNA and appropriate forward and reverse primers. The following primers were used: Bax, F:5'-AGAGGATGATTGCCGCCGT-3' and R:5-CAACCACCCTGGTCTTGGATC-3' [30]; Bcl2, F:5'-CATGTGTGTGGAGAGCGTCAA -3' and R:5'-ACAGTTCCACAAAGGCATCCC-3' [30]; Bad, F:5'-CCGAGTGAGCAGGAAGACTC-3' and R: 5'-GGTAGGAGCTGTGGCGACT-3' [38]; ApoE, F:5'-TGCTCAGCTCCCAGGTCAC-3' and R:5'-GCCTTCAACTCCTTCATGGTCT-3' [39]; SepP, F:5'-GTCTTCCCTCAGTAAGTACT-3' and R:5'-CTTCTCCACATTGCTGAGGT-3' [24]; Nrf2, F:5'-TGAAGCTCAGCTCGCATTGATCC-3' and R:5'-AAGATACAAGGTGCTGAGCCGCC-3' [17]; GAPDH, F:5'-TCCTCTGACTTCAACAGCGACAC-3' and R:5'-CACCCTGTTGCTGTAGCCAAATTC-3' [40]. The qRT-PCR assays were performed on the StepOnePlus Real-Time PCR System (Life Technologies Corp.). The samples were initially denatured at 95°C for 20 seconds followed by PCR amplification at 95°C for 3 seconds and 62°C for 30 seconds for 40 cycles. Purity of product was checked using the melt curve analysis program which was defined as 95°C for 15 seconds, 60°C for 1 min and 95°C for 15 seconds with temperature increments of 0.3°C. Relative quantification was analyzed using GAPDH as endogenous control and ΔCT algorithm using StepOne Software v2.2 RQ Study (Life Technologies Corp.).

### Statistical analysis

The data were analyzed by our statistician collaborators Thomas Havighurst and KyungMann Kim using multiple statistical methods. Briefly, tumor volume was recorded at three times: pre-treatment, and one and two week post-treatment. Log-transformation was found to be the most part appropriate test. Statistical analyses on tumor data and serum PSA level were performed with ANCOVA (Analysis of Covariance) models, examining treatment as a two-way and one-way effect. The two-way ANCOVA analysis did not show significant interaction between MSA and γT. Therefore, the data were analyzed with one-way design considering treatment groups as a single factor. Dunnett's multiple comparison tests, in which only comparisons with controls are considered, was used for post-hoc analysis. Mean data of 10 mice were represented with ± standard errors for each estimated mean.

The densitometry data of immunoblots were analyzed using one-way repeated measures ANOVA followed by Dunnett's test. The qRT-PCR data were analyzed using StepOne Software v2.2 RQ Study (Life Technologies Corp.), and CT (threshold cycle) value was used for further statistical analysis. CT value is the cycle number at which the fluorescence generated within a reaction crosses the threshold line. The higher CT cycle is associated with less cDNA in the sample at the outset. For the purposes of testing differences, the quantity of interest to be compared between treatment groups is the difference between the CT value of target gene (the gene we are interested in testing) and the CT value of the endogenous control gene (the gene used to normalize the results). This difference (known as ΔCT) was modeled and tested by treatment group. It should be noted that a smaller ΔCT corresponds to an increased gene expression. Similar to immunoblot data, ANOVA followed by Dunnett's test was used to calculate statistical differences.
